# ﻿New species, new records and common species of Pluteussect.Celluloderma from northern China

**DOI:** 10.3897/mycokeys.104.117841

**Published:** 2024-04-16

**Authors:** Zheng-Xiang Qi, Ke-Qing Qian, Lei Yue, Li-Bo Wang, Di-Zhe Guo, Dong-Mei Wu, Neng Gao, Bo Zhang, Yu Li

**Affiliations:** 1 Engineering Research Center of Edible and Medicinal Fungi, Ministry of Education, Jilin Agricultural University, Changchun, Jilin 130118, Changchun, China Jilin Agricultural University Changchun China; 2 Biotechnology Research Institute, Xinjiang Academy of Agricultural and Reclamation Sciences, Shihezi 830011, China Biotechnology Research Institute, Xinjiang Academy of Agricultural and Reclamation Sciences Shihezi China

**Keywords:** Line drawings, morphology, phylogeny, wood-rotting fungi

## Abstract

Wood-rotting fungi are organisms that can decompose wood substrates and extract nutrients from them to support their growth. They play a crucial role in the material cycle of forest ecosystems. The genus *Pluteus* plays a significant role in wood decomposition. In this study, the morphology and molecular systematics of the sect. Celluloderma of the genus *Pluteus* were carried out. *Pluteusbrunneodiscus* was identified as a new species, along with the discovery of two new records, *P.cystidiosus* and *P.chrysophlebius*, and a common species, *P.romellii*. *Pluteusbrunneodiscus* is characterized by the brown center of the pileus that transitions to white towards the margins, with the surface cracking to form irregular granules. It is typically found in Populus forests growing on decomposing twigs or wood chips. Line drawings, color photographs, and phylogenetic analyses of related species within the genus *Pluteus* accompany the descriptions of these four species. The analyses are based on ITS + TEF1-α sequence data. Finally, a key for the twenty species within the sect. Celluloderma of the genus *Pluteus*, which has been documented in China, is provided.

## ﻿Introduction

The genus *Pluteus* Fr., which belongs to the Basidiomycota, Agaricomycetes, Agaricales, Pluteaceae, was established by Fries in 1863. The genus *Pluteus* is characterized by its free lamellae, pinkish spore print, inverse hymenophoral trama, smooth spherical to ellipsoidal basidiospores, various forms of pleurocystidia, and often cheilocystidia. It is predominantly found on decaying wood and has a global distribution (Vellinga and Schreurs 1985; Singer 1986; Justo et al. 2011a, 2011b).

The genus *Pluteus* was categorized into three sections based on the characteristics of the cystidia and pileipellis *viz.* (1) sect. Pluteus Fr is characterized by the existence of a cutis pileipellis and thick-walled pleurocystidia, (2) sect. Hispidoderma Fayod is characterized by a pileipellis that is a trichoderm composed of elongated cells and thin-walled pleurocystidia and (3) sect. Celluloderma Fayod is characterized by a pileipellis that is a hymeniderm or hymeniderm with cystidioid elements, comprising of clavate to spheropedunculate cells and thin-walled pleurocystidia (Lange 1917; Imai 1938; Singer 1956). Molecular phylogenetic analysis provides support for three sections (*Pluteus* Fr, *Hispidoderma* Fayod, and *Celluloderma* Fayod) (Menolli et al. 2010; Justo et al. 2011a, 2011b).

Singer further subdivided Pluteu s sect. Celluloderma into two subsections based on the composition of the pileipellis: subsect. Mixtini Singer, is characterized by elongated elements, and subsect. Eucellulodermini Singer is characterized by the absence of such elements (Singer 1956; Singer 1958). The molecular phylogenetic studies do not divide the Pluteussect.Celluloderma into two subsections (Justo et al. 2011b). Some species belonged to the sect. Celluloderma (e.g., *P.ephebeus* (Fr.) Gillet and related species). Based on their characteristics, species composed of non-metuloid cystidia and a pileipellis as cutis should belong to the sect. Hispidoderma. This is not consistent with the phylogenetic results. Thus, the classification of the two subsections of sect. Celluloderma needs to be further justified.

Vellinga and Schreurs (1985) proposed a different taxonomic system to distinguish these species (e.g., *P.ephebeus* (Fr.) Gillet and related species), dividing the Pluteussect.Celluloderma into three subsects, *Mixtini*, *Eucelullodermini*, and *Hispidodermini* (Fayod) Vellinga and Schreurs. The latter is characterized by a trichodermal pileipellis or a euhymeniderm consisting of cylindrical to fusiform elements, which are similar to some of the characteristics of the sect. Hispidoderma. Additionally, Schreurs and Vellinga proposed a new group sect. Villosi Schreurs and Vellinga, containing species with a cutis-like pileipellis and non-metuloid (Singer 1958; Singer 1986). The proposed new sections and subsections by Singer (1958, 1986), Vellinga, and Schreurs (1985) lack support from molecular systematic studies (Justo et al. 2011a; Justo et al. 2012).

Recent studies (Minnis et al. 2006; Menolli et al. 2010; Justo et al. 2011a, 2011b; Vizzini and Ercole 2011) have indicated that sect. Celluloderma includes species characterized by the presence of non-metuloid pleurocystidia and a pileipellis that is either euhymeniderm or epithelioid hymeniderm, composed of short elements, which may or may not be intermixed or not with elongate cystidioid elements (corresponding to Pluteussect.Celluloderma as defined by Singer 1956, 1958, 1986), refers to species with a cutis-like pileipellis and non-metuloid cystidia (corresponding to Pluteussect.Villosi or *Hispidoderma* sensu Singer p.p.).

In the current investigation, a new species, (*P.brunneodiscus*), two new records to China, (*P.chrysophlebius* and *P.cystidiosus*), and a common species, (*P.romellii*) are described. Detailed descriptions and illustrations are provided for the four species, along with clarification of the phylogenetic relationships of the identified species and related taxa from the genus Pluteussect.Celluloderma.

## ﻿Materials and methods

### ﻿Morphology

In the field, photographs of fresh basidiomata were taken to scientifically and adequately reflect the growing environment and characteristics of the basidiomata, including the shape of the pileus, the color of the lamellae, and Munsell Soil Color Chart was followed for color codes (Munsell 2009). For fresh basidiomata, we promptly determined the size and recorded in detail the shape, size, color, odor, and other macroscopic characteristics of the basidiomata pileus, lamellae, and stipes. About 15 g of fresh context and lamellae were dried in a Ziplock bag with silica gel and returned to the lab for DNA extraction. Fresh basidiomata were dried at 40 ~ 45 °C (Hu et al. 2022), using a plant drying oven and preserved in the fungarium of Jilin Agricultural University (FJAU).

The observation of microstructural features was based on dried specimens. The dry specimens were rehydrated in 94% ethanol for microscopic examination and then mounted in 3% potassium hydroxide (KOH), 1% Congo Red, and Melzer’s Reagent, using a light microscope (ZEISS, DM1000, Oberkochen, Germany). Specifically, the following symbols were used in the description: [n/m/p] indicates that ‘n’ randomly selected basidiospores from ‘m’ basidiomata of ‘p’ collections were measured, ‘avl’ means the average length of basidiospores, except for the extreme values, ‘avw’ refers to the average width of the basidiospores, except the extreme values, ‘Q’ represents the quotient of the length and width of a single basidiospore inside view, ‘Qm’ refers to the average Q value of all basidiospores ± standard deviation. The dimensions for basidiospores are given as (a)b–c(d). The range of b–c contains a minimum of 90% of the measured values. Extreme values (i.e., a and b) are given in parentheses (Qi et al. 2022).

### ﻿Molecular phylogeny

#### ﻿DNA extraction, PCR amplification, and sequencing

According to the instructions, the total DNA of the specimens was extracted by the new plant genomic DNA extraction kit from Jiangsu Kangwei Century Biotechnology Limited Company, P.R. China. Subsequently, sequences of the internal transcribed spacer (ITS) region, and translation elongation factor 1-α (TEF1-α) were used for phylogenetic analyses. The amplification primers of the nr ITS: ITS1–5.8S–ITS2 regions were ITS1F and ITS4/ITS4B (White et al. 1990), and TEF1-α regions were EF1–983F and EF1–1567R (Rehner and Buckley 2005). The amplification reactions were carried out in a 25 µL system. The total amount of PCR mixed was as follows: dd H_2_O 13.5 µL, 10 × Taq Buffer 5 µL, 10 mM dNTPs 1 µL, 10 mM upstream primer 1 µL, 10 mM downstream primer 1 µL, DNA sample 2 µL, 2 U/mm Taq Polymerase 1.5 µL. The cycle parameters were as follows: 5 min at 98 °C; 30 s at 98 °C, 30 s at 55 °C, 1 min at 72 °C for 40 cycles; 7 min at 72 °C; storage at 4 °C (Ševcíková et al. 2022). The PCR product was subjected to 1% agarose gel electrophoresis. The purified PCR products were sent to Sangon Biotech Limited Company, P.R. China for sequencing using the Sanger method. The sequencing results were clipped with Seqman 7.1.0 (Swindell and Plasterer 1997) and subsequently deposited in GenBank (https://www.ncbi.nlm.nih.gov/genbank).

### ﻿Data analysis

The species that were morphologically similar to new species, newly recorded species, and common species, and have high sequence similarity after blast were selected (Justo et al. 2011b, 2012; Menolli et al. 2015; Desjardin and Perry 2018; Hosen et al. 2019; Hosen et al. 2021; Ševčíková et al. 2022; Qi et al. 2022; Malysheva et al. 2023; Ševcíková et al. 2023; Xu et al. 2023), and details of the ITS and TEF1-α sequences of these species are shown in Table 1. The ITS and TEF1-α dataset comprised 134 representative sequences that exhibited the highest similarity to *Pluteus* spp., and two sequences of *Volvopluteusmichiganensis* (A.H. Sm.) Justo and Minnis. as an outgroup.

**Table 1.** Names, collection numbers, reported countries and corresponding GenBank accession numbers of the taxa used in this study.

Taxon	Collection	Country	GenBank No.		Reference
			ITS	TEF1-α	
*Pluteusabsconditus*	iNaturalist 112240775	USA (TN)	OR229047	OR242143	Ševcíková et al. 2023
*P.absconditus*	MO 136488	USA (TN)	KM983689	OR242144	Ševcíková et al. 2023
P.aff.ephebeus	BPI 882530	USA-Illinois	JQ065025	–	Menolli et al. 2015
P.aff.ephebeus	BPI 882531	USA-Illinois	JQ065026	–	Menolli et al. 2015
P.aff.ephebeus	HHB1213	USA-New Mexico	KM983670	–	Menolli et al. 2015
P.aff.ephebeus	AJ478	USA-Vigin Islands	KM983675	–	Menolli et al. 2015
P.aff.ephebeus	AJ535	Dominican Republic	KM983676	–	Menolli et al. 2015
*P.aletaiensis*	HMJAU 60207	China	OM991943	OP573273	Qi et al. 2022
*P.aletaiensis*	HMJAU 60208	China	OM992247	OP573274	Qi et al. 2022
*P.aurantiorugosus*	GDGM41547	China	MK791275	–	Ševcíková et al. 2022
*P.aurantiorugosus*	LE 312815	Russia (Europe)	ON864103	ON813296	Ševcíková et al. 2022
*P.austrofulvus*	AJ 857	USA, Arkansas	KM983701	ON813290	Ševcíková et al. 2022
*P.austrofulvus*	AJ 860	USA, Arkansas	KM983699	ON813288	Ševcíková et al. 2022
*P.brunneidiscus*	HMJAU 60206	China	OM991893	–	Qi et al. 2022
*P.brunneidiscus*	HMJAU 60210	China	OM943513	–	Qi et al. 2022
*P.cervinus*	REG 13641	USA	HM562152	–	Qi et al. 2022
P.cf.nanus	LE 213093	Russia	FJ774081	–	Justo et al. 2011
P.cf.ephebeus	LOU15198	Spain	KM983671	–	Menolli et al. 2015
P.cf.ephebeus	Shaffer4673	France	HM562080	–	Menolli et al. 2015
P.cf.ephebeus	Pearson sn	England	HM562198	–	Menolli et al. 2015
P.cf.ephebeus	9823	Italy	JF908620	–	Menolli et al. 2015
P.cf.ephebeus	10151	Italy	JF908621	–	Menolli et al. 2015
P.cf.fastigiatus	NKI12	Brazil	KM983678	–	Menolli et al. 2015
P.cf.fuliginosus	FK2158	Brazil	KM983677	–	Menolli et al. 2015
*P.chrysophlebius*	TNSF12383	Japan	HM562125	–	Justo et al. 2011a
*P.chrysophlebius*	SF10 (BPI)	USA (IL)	HM562180	–	Justo et al. 2011a
*P.chrysophlebius*	TNSF12388	Japan	HM562088	–	Justo et al. 2011a
*P.chrysophlebius*	SF12 (BPI)	USA (IL)	HM562182	–	Justo et al. 2011a
*P.chrysophlebius*	SF11 (SIU)	USA (IL)	HM562181	–	Justo et al. 2011a
***P.chrysophlebius***	**FJAU66561**	**China**	**OR994065**	**PP062824**	This study
*P.cutefractus*	BRNM825872	Spain	OR229050	OR242162	Ševcíková et al. 2023
*P.cutefractus*	GM 3458	Spain	OR229048	OR242165	Ševcíková et al. 2023
*P.cutefractus*	FG 26092015	Slovenia	OR229053	OR242164	Ševcíková et al. 2023
*P.cystidiosus*	LE 312852	Russia (Far East)	OR229063	OR242175	Ševcíková et al. 2023
*P.cystidiosus*	LE 313335	Russia (Far East)	OR229062	OR242174	Ševcíková et al. 2023
*P.cystidiosus*	AJ 782 (NBM-F-009790)	USA (MA)	KM983687	OR242171	Ševcíková et al. 2023
*P.cystidiosus*	AJ 617 (NBM-F-009788)	USA (NY)	KM983686	OR242173	Ševcíková et al. 2023
***P.cystidiosus***	**FJAU66556**	**China**	**OR994068**	PP062825	This study
***P.cystidiosus***	**FJAU66557**	**China**	**PP002166**	**PP062826**	This study
*P.diptychocystis*	NMJ184	Brazil	KM983674	–	Menolli et al. 2015
*P.ephebeus*	AJ234	Spain	HM562044	–	Menolli et al. 2015
*P.fenzlii*	TNSF12376	Japan	HM562091	–	Menolli et al. 2015
*P.fenzlii*	F1020647	Slovakia	HM562111	–	Menolli et al. 2015
*P.fenzlii*	LE 246083	Russia	FJ774082	–	Holec et al. 2017
*P.fulvibadius*	AJ 815	USA, California	KM983698	ON813285	Ševcíková et al. 2022
*P.fulvibadius*	HRL3391	Canada, Québec	ON864094	ON813287	Ševcíková et al. 2022
*P.gausapatus*	BRNM817745	South Korea	OR229067	OR242177	Ševcíková et al. 2023
*P.gausapatus*	BRNM817745	South Korea	OR229067	OR242177	Ševcíková et al. 2023
*P.halonatus*	FK2084	Brazil	KM983680	–	Menolli et al. 2015
*P.halonatus*	NKI17	Brazil	KM983679	–	Menolli et al. 2015
*P.heteromarginatus*	AJ172	USA	HM562058	–	Hosen et al.2019
*P.hirtellus*	SFSU:DED 8259	West Africa	MG968804	–	Desjardin and Perry 2018
*P.inconspicuus*	PDD 72485	New Zealand	MN738614	–	Ševcíková et al. 2023
*P.inflatus*	BRNM817761	Czech Republic	OR229033	OR242136	Ševcíková et al. 2023
*P.inflatus*	BRNM825836	Czech Republic	OR229035	OR242132	Ševcíková et al. 2023
*P.inflatus*	BRNM825837	Czech Republic	OR229036	OR242133	Ševcíková et al. 2023
*P.insidiosus*	15120	Italy	JF908626	–	Justo et al. 2012
*P.longistriatus*	Minnis309203	USA	HM562082	–	Hosen et al.2019
*P.lucidus*	LE F-347426	Russia	OQ732746	–	Malysheva et al. 2023
*P.mammillatus*	Singer244A	USA-Florida	HM562120	–	Holec et al. 2017
*P.mammillatus*	Minnis309202	USA-Missouri	HM562086	–	Holec et al. 2017
*P.mammillatus*	ASM7916	USA-Missouri	HM562119	–	Holec et al. 2017
***P.brunneodiscus***	**FJAU66132**	**China**	**PP002168**	PP062821	This study
***P.brunneodiscus***	**FJAU66133**	**China**	**PP002169**	**PP062822**	This study
***P.brunneodiscus***	**FJAU66134**	**China**	**PP002167**	**PP062823**	This study
*P.parvisporus*	AJ 855	USA, Arkansas	ON864099	ON813295	Ševcíková et al. 2022
*P.parvisporus*	iNaturalist 112236342	USA, Tennessee	ON864098	ON813294	Ševcíková et al. 2022
*P.phlebophorus*	AJ 81(NBM-F-009110)	Spain	HM562039	ON133554	Ševcíková et al. 2023
*P.phlebophorus*	AJ228 (LOU)	Spain	HM562138	–	Justo et al. 2011a
*P.phlebophorus*	AJ194 (LOU)	Spain	HM562137	–	Justo et al. 2011a
*P.phlebophorus*	AJ193 (LOU)	Spain	HM562144	–	Justo et al. 2011a
*P.plautus*	P59	USA-California	KF306016	–	Menolli et al. 2015
*P.podospileus*	LE 303682	Russia (South Siberia)	KX216331	OR242169	Ševcíková et al. 2023
*P.podospileus*	LE 303687	Russia (South Siberia)	KX216332	OR242168	Ševcíková et al. 2023
*P.podospileus*	LE 313589	Russia (South Siberia)	OR229060	OR242167	Ševcíková et al. 2023
P.riberaltensisvar.conquistensis	FK1043	Brazil	HM562162	–	Menolli et al. 2015
*P.romellii*	AJ 232	Spain	HM562062	ON813280	Ševcíková et al. 2022
*P.romellii*	BRNM 761731	Czech Republic	ON864065	ON813278	Ševcíková et al. 2022
*P.romellii*	BRNM 816205	Czech Republic	ON864063	ON813276	Ševcíková et al. 2022
*P.romellii*	BRNM 825845	Slovakia	ON864070	ON813281	Ševcíková et al. 2022
***P.romellii***	**FJAU66558**	**China**	**OR994057**	**PP062827**	This study
***P.romellii***	**FJAU66559**	**China**	**OR994061**	**PP062828**	This study
*P.rugosidiscus*	BRNM761706	Slovakia	MH010876	LT991752	Ševcíková et al. 2023
*P.rugosidiscus*	Homola109 (MICH)	USA (MI)	HM562079	–	Justo et al. 2011a
*Pluteus* sp.	SP394389	USA	HM562161	–	Justo et al. 2012
*Pluteus* sp.	iNaturalist 27406926 (NBM-F-009806)	USA (IN)	ON006984	OR242176	Ševcíková et al. 2023
*P.squarrosus*	GDGM 42320	China	MK791274	–	Hosen et al.2019
*P.squarrosus*	GDGM 42302	China	MK791273	–	Hosen et al.2019
*P.thomsonii*	LE 303662	Russia	KX216329	–	Justo et al. 2012
*P.tomentosulus*	MO163564	USA-Pennsylvania	KM983673	–	Menolli et al. 2015
*P.tomentosulus*	MO93719	USA-Oregon	KM983672	–	Menolli et al. 2015
*V.michiganensis*	HMJAU-CR45	China	MW242665	–	Qi et al. 2022
*Volvopluteusmichiganensis*	HMJAU-CR43	China	MW242664	–	Qi et al. 2022

Bold fonts are the sequences to be determined in this study.

For obtaining ITS + TEF1-α datasets of related species, sequence alignment was initially performed for ITS and TEF1-α using the “automatic” strategy and normal alignment mode of MACSE V2.03 (Ranwez et al. 2018) and MAFFT (Katoh and Standley 2013), respectively. Subsequently, the alignments were manually adjusted in BioEdit v7.1.3 (Hall 1999). Afterward, ITS and TEF1-α sequences were aligned and combined using Phylosuit V1.2.2 (Zhang et al. 2020). Then, ModelFinder (Kalyaanamoorthy et al. 2017) was used to select the best-fit models using the Bayesian information criterion (BIC). In this case, the Maximum likelihood (ML) analyses were performed in IQTree 1.6.8 (Nguyen et al. 2015), and the Bayesian inference phylogenies were performed in MrBayes 3.2.6 (Ronquist et al. 2012) (two parallel runs, 2,000,000 generations), in which the initial 25% of sampled data were discarded as burn-in. The above software was integrated into PhyloSuite 1.2.2 (Zhang et al. 2020). The ML phylogenetic tree was evaluated using the bootstrap method with a bootstrap value of 1,000 replicates; BI determined that the analysis reached smoothness with a variance of less than 0.01 and terminated the calculation. Finally, the evolutionary tree was followed up with Figtree v1.4.

## ﻿Results

### ﻿Phylogenetic analyses

This study’s nrITS dataset comprises 93 sequences and 650 characters (gaps included). The TEF1-α dataset comprises 41 sequences and 530 characters (gaps included). The combined nrITS + TEF1-α dataset consists of 134 sequences and 1180 characters, including gaps. Of these, 16 sequences (8 nrITS and 8 TEF1-α) were newly generated in this study (Table 1). The overall topologies of the ML and BI trees were nearly identical for all datasets.

**Figure 1.**
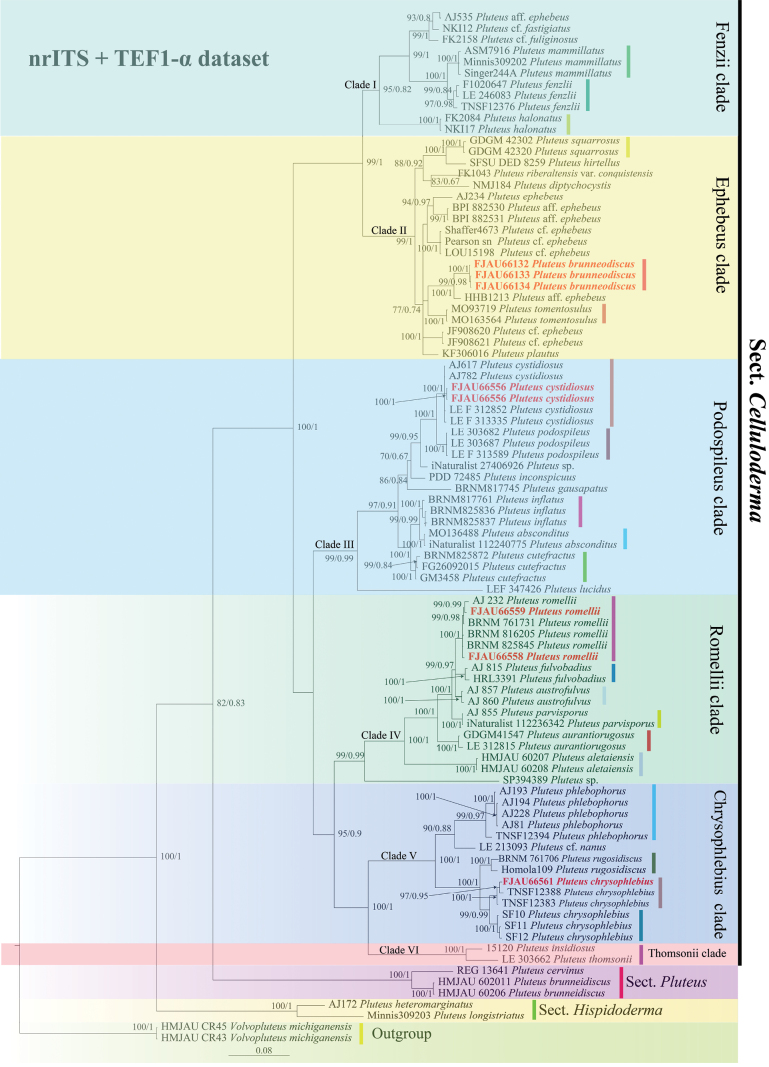
Phylogenetic tree of the sect. Celluloderma of the genus *Pluteus*. The best tree from the ML and BI analysis of the nrITS + TEF1-α dataset. The two values of internal nodes respectively represent the maximum likelihood bootstrap (MLBP)/Bayesian posterior probability (BIPP). This study species is in bold and red font.

For clarity and brevity, we use the term “strongly supported” for a clade/relation that receives a bootstrap (BS) 90 and a posterior probability (PP) = 1, and “well supported” if it receives a BS 70 and a PP of 0.95. The individual support values are shown in Fig. 1.

Within the sect. Celluloderma, six strongly supported clades are recovered in the combined nrITS + TEF1-α dataset:

1. Clade I: This includes the clade we consider to represent *P.mammillatus* (Longyear) Minnis, Sundb. & Methven from the USA, *P.fenzlii* (Schulzer) Corriol & P.-A. Moreau from Japan, Slovakia, and Russia, *P.halonatus* from Brazil.
2. Clade II: Includes only the newly described *P.brunneodiscus* from China. This also includes the clade we consider to represent *P.squarrosus* Hosen & T.H. Li from China, *P.hirtellus* Desjardin & B.A. Perry from West Africa, *P.plautus* (Weinm.) Gillet from the USA, *P.tomentosulus* Peck from the USA, *P.diptychocystis* Singer from Brazil, and *P.riberaltensis* var. *conquistensis* from Brazil, while *P.ephebeus* from Spain, France, England, and Italy (*P.* cf. *ephebeus* and *P.* aff. *ephebeus*), *P.fuliginosus* Murrill from Brazil (*P.* cf. *fuliginosus*), *P.fastigiatus* Singer from Brazil (*P.* cf. *fastigiatus*).
3. Clade III: Includes the newly described *P.cystidiosus* (China). This clade also includes the clade we consider to represent *P.podospileus* Sacc. & Cub. (Russia), *P.cutefractus* Ferisin, Dovana & Justo (Spain, Slovenia), *P.inflatus* Velen (Czech Republic), *P.inconspicuus* E. Horak (New Zealand); three recently described species, *P.cystidiosus* (Russia, USA), *P.absconditus* Justo, Kalichman & S.D. Russell (USA), and *P.gausapatus* Ševčíková & Antonín (South Korea), and one likely undescribed species from the USA (iNaturalist 27406926).
4. Clade IV: Includes the newly described *P.romellii* (China). It also includes *P.fulvibadius* Murrill (USA and Canada), *P.aurantiorugosus* (Trog) Sacc (China and Russia). Three recently described species, *P.austrofulvus* Justo, Minnis, S.D. Russell & Kalichman (USA), *P.parvisporus* Justo, Kalichman & S.D. Russell (USA) and *P.aletaiensis* Z.X. Qi, B. Zhang and Yu Li (China).
5. Clade V: Includes the newly described *P.chrysophlebius* (China). This clade also includes the clade we consider to represent *P.chrysophlebius* (Japan, USA, Japan), *P.phlebophorus* (Ditmar) P. Kumm (Spain), and *P.rugosidiscus* Murrill (Slovakia, USA).
6. Clade VI: This clade includes the clade that we consider to represent *P.insidiosus* Vellinga & Schreurs (Italy) and *P.thomsonii* (Berk. & Broome) Dennis (Russia).

## ﻿Taxonomy

### ﻿ Pluteus brunneodiscus

Taxon classificationFungiAgaricalesPluteaceae

Z.X. QI, B. Zhang & Y. Li sp. nov.

B5E3E75A-A81F-51C6-8642-CDDC18D9ABE5

851479

Figs 2A–B , 3

#### Typification.

China. Xinjiang Uygur Autonomous Region, Ili Kazakh Autonomous Prefecture, Tekes County, Aktamu Wetland, 43°15'22.61"N, 81°75'90.21"E, alt. 1243 m, 6 July 2022, Z.X. Qi (FJAU 66134, holotype!).

#### Sequences holotype.

ITS: PP002167, TEF1-α: PP062823.

#### Etymology.

“brunneo-”: brown, “-discus”: pileus disc. The species epithet “brunneodiscus” (Lat.) refers to the brown of the middle part of the pileus disc.

#### Diagnosis.

*Pluteusbrunneodiscus* differs from *P.tomentosulus* by its brown pileus in the middle, transitioning to white toward the margins, and the surface cracks to form irregular granules. It grows in poplar forests (Populusalbavar.pyramidalis Bge) with decaying wood branches or chips.

#### Description.

Basidiomata medium to large. Pileus 39–71 mm in diam, initially compressed hemispherical, surface with dense brown irregular granules (5.0YR 5/2), dirty white (5.0YR 9/2), middle brown (5.0YR 4/4), margin entire, gradually spreading at maturity, pileus middle dark brown (5.0YR 3/6), margin irregularly dehiscent at maturity or after hygrophanous. Context whitish (5.0YR 9/2), odorless, 3–6 mm thick. Lamellae initially dirty white (5.0YR 9/2), becoming flesh-brown to earth-brown at maturity (5.0YR 8/4- 5.0YR 6/4), free, dense, thick, unequal, slightly ventricose, 6–7 mm wide. Stipe 37–55 mm long, 8–11 mm wide, dirty white (5.0YR 9/2), cylindrical, slightly thicker at the base, fibrous, with white longitudinal stripes on the surface. Odorless. Spore prints pink.

Basidiospores [120, 12, 3] (–6.5) 7.0–7.5 (–8.0) × 5.0–6.0 (–6.5) µm, avL × avW = 7.0 × 6.0 µm, Q = 1.16–1.30–1.45 µm, avQ = 1.16 µm, globose, subglobose, slightly pink, smooth, thin-walled, non-dextrinoid, partially containing one droplet or irregular inclusions. Basidia 25–32 × 7–11 μm, fusiform to clavate, thin-walled, 4–sterigmate, and hyaline in KOH. Pleurocystidia abundant, scattered, 55–102 × 22–36 μm, vesicular to narrowly vesicular, or clavate, thin-walled, smooth, and hyaline in KOH. Cheilocystidia abundant, clustered, 41–79 × 18–29 μm, subfusiform to fusiform, or ventrally bulbous, apically broadly digitate 15–23 μm long, thin-walled, hyaline. Lamellar trama divergent. Pileipellis a cutis to trichodermium, hyphae 4–10 µm diam, cylindrical, hyaline, non-gelatinous; terminal cells inflated, 62–91 × 22–31 μm, obtusely rounded or pointed apically, thin-walled, with brown cytoplasmic pigments. Stipitipellis a cutis, hyphae 5–9 µm diam, cylindrical, hyaline, non-incrusted, non-gelatinous, thin-walled. Caulocystidia absent. Clamp connections absent in all tissues.

**Figure 2.**
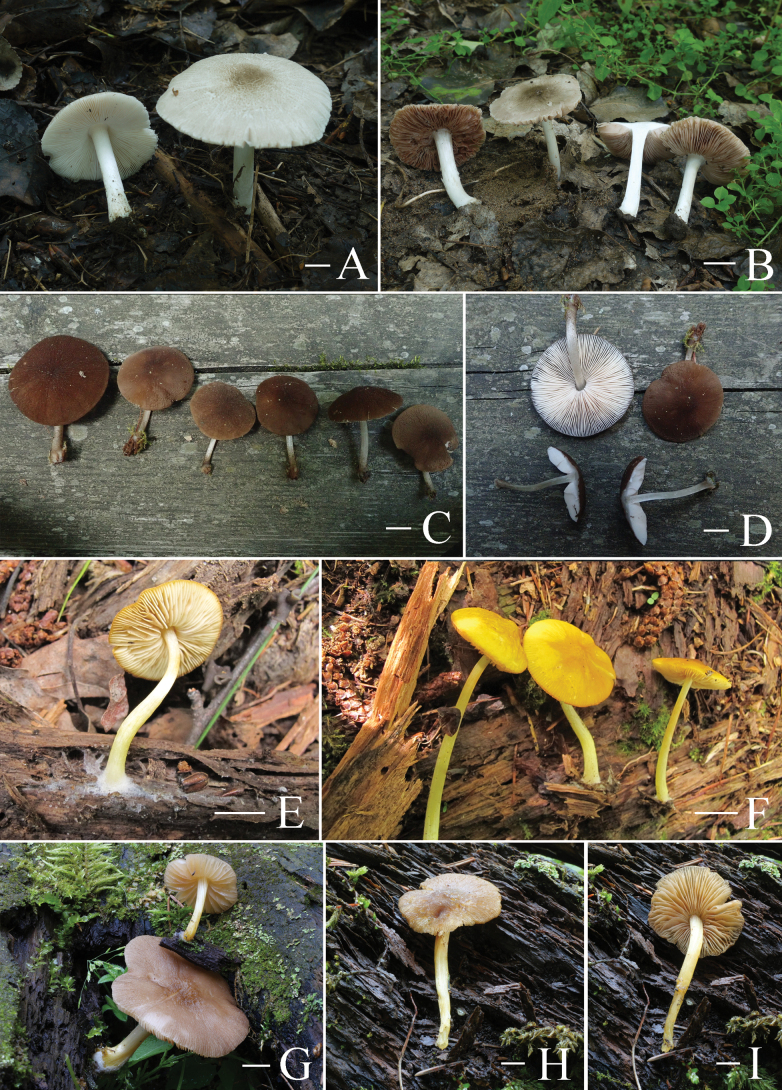
Basidiomata features **A–B***Pluteusbrunneodiscus***C–D***P.cystidiosus***E–F***P.chrysophlebius***G–I***P.romellii*. Photos by Zheng-xiang Qi (**A–B, G–I**). Photos by Di-zhe Guo (**C–F**). Scale bars: 1 cm.

#### Ecology and distribution.

Solitary to scattered on the ground in the broad-leaved forests (Populusalbavar.pyramidalis Bge) with decaying wood branches or wood chips. Known from Xinjiang Uygur Autonomous Region of China.

#### Additional specimens examined.

China. Xinjiang Uygur Autonomous Region, Ili Kazakh Autonomous Prefecture, Tekes County, Aktamu Wetland, 43°15'22.61"N, 81°75'90.21"E, alt. 1243 m, 6 July 2022, Z.X. Qi, D.M. Wu, N. Gao and B.K. Cui, FJAU 66132 (ITS: PP002168, TEF1-α: PP062821). China. Xinjiang Uygur Autonomous Region, Ili Kazakh Autonomous Prefecture, Tekes County, Aktamu Wetland, 43°15'22.61"N, 81°75'90.21"E, alt. 1243 m, 6 July 2022, Z.X. Qi, FJAU 66133 (ITS: PP002169, TEF1-α: PP062822).

#### Notes.

Morphologically, *Pluteusbrunneodiscus* is very similar to *P.tomentosulus* in having a white pileus. The difference lies in the surface texture, as *P.tomentosulus* has a very finely granular-tomentose surface that becomes bald at maturity, while *P.brunneodiscus* features a brown center of the pileus, transitioning to white toward the margins, with the surface cracking to form irregular granules (Vellinga and Schreurs 1985; Orton 1986; Vellinga 1990; Desjardin and Perry 2018).

In phylogenetic analyses, *P.brunneodiscus* clusters in the ephebeus clade as a sister species to P.aff.ephebeus, and has a support ratio of 1/100. However, the pileus of P.aff.ephebeus are sooty, shield-shaped fruiting bodies with pubescent or downy surfaces. They grow on rotting wood or stumps and are widely distributed in Britain and Ireland (Orton 1986; Justo et al. 2011a; Menolli et al. 2015). These characteristics distinguish *P.brunneodiscus* from P.aff.ephebeus.

**Figure 3.**
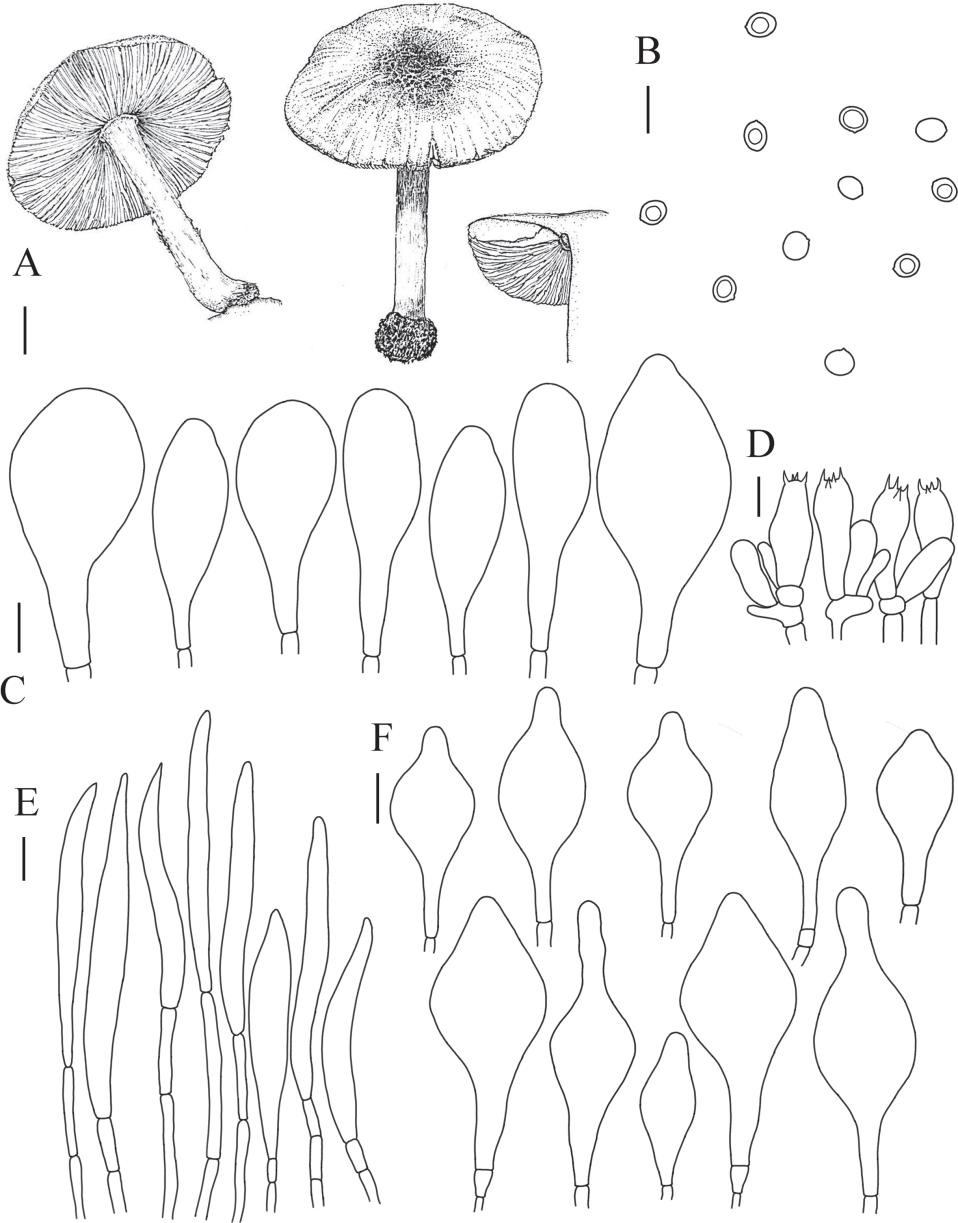
**A** Macroscopic characteristics of *Pluteusbrunneodiscus***B** basidiospores **C** pleurocystidia **D** basidia **E** pileipellis terminal cells **F** cheilocystidia. Scale bars: 1 cm (**A**); 10 µm (**B–F**).

### ﻿ Pluteus cystidiosus

Taxon classificationFungiAgaricalesPluteaceae

(Minnis and Sundb.) Justo, Malysheva & Lebeuf, in Ševčíková et al., Journal of Fungi 9(9, no. 898): 34 (2023)

52AA96E6-A260-50AB-BA5F-B372295DC1A7

Figs 2C–D , 4

Pluteus seticeps var. cystidiosus Minnis and Sundberg N. Amer. Fung. 5(1): 13 (2010). Syn.

#### Description.

Basidiomata medium to large. Pileus 25–41 mm in diam, compressed hemispherical, surface spreading when young, surface with longitudinal vein-like folds from middle to margin when mature, margin mostly transverse folds, light brown to dark brown (5.0YR 5/6-5.0YR 4/12), margin entire. Context dirty white (2.5YR 9/4), odorless, 5–8 mm thick. Lamellae dirty white (2.5YR 9/4), free, dense, thick, unequal, ventricose, 15–18 mm wide. Stipe 30–41 mm long, 12–17 mm wide, cylindrical, slightly thicker at the base, hollow, fibrous, with brown serpentine or crumbly scales on the surface (2.5YR 9/2). Odorless. Spore prints pink.

Basidiospores [200, 10, 2] (–5.0) 5.5–6.0 (–6.5) × (–4.5) 5.0–5.5 μm, avL × avW = 6.0 × 5.0 µm, Q = 1.10–1.20–1.30 μm, avQ = 1.20 μm, spherical, subglobose, slightly pink, smooth, thin-walled, non-dextrinoid, partially containing one droplet or irregular inclusions. Basidia 23–31 × 7–10 μm, clavate, thin-walled, 4-sterigmate, and hyaline in KOH. Pleurocystidia abundant, scattered, 55–102 × 22–36 μm, rod-shaped or subpyriform, vesicular, thin-walled, smooth, and hyaline in KOH. Cheilocystidia abundant, clustered, 37–60 × 15–22 μm, clavate, fusiform or vesicular, thin-walled. Lamellar trama divergent. Pileipellis a hymeniderm or epithelioid hymeniderm, made up of two types of elements; spheropedunculate or pyriform, 27–55 × 24–34 μm; broadly fusiform, inﬂated-fusiform, lanceolate, narrowly utriform, often mucronate, 56–105 × 11–23 μm; all elements with brown intracellular pigment, often aggregated in spots, slightly thick-walled. Stipitipellis a cutis of cylindrical, hyphae 8–11 μm wide, with pale brown pigment. Caulocystidia common, often in clusters, 36–112 × 9–20 μm, cylindrical, narrowly clavate, narrowly fusiform, spheropedunculate, with brown or yellow-brown pigment. Clamp connections absent in all studied tissues.

#### Ecology.

Scattered on decaying wood in mixed coniferous forests (*Pinuskoraiensis* Siebold and Zucc).

#### Distribution.

Canada, the USA, Japan, Russian Far East.

#### Additional specimens examined.

China. Heilongjiang Province, Liangshui National Nature Reserve. 47°11'22.24"N, 128°47'89.11"E, 23 June 2019, D.Z. Guo, FJAU 66556 (ITS: OR994068, TEF1-α: PP062825). China. Heilongjiang Province, Liangshui National Nature Reserve. 47°11'22.24"N, 128°47'89.11"E, 28 June 2019, D.Z. Guo, FJAU 66557 (ITS: PP002166, TEF1-α: PP062826).

**Note.**Ševcíková et al. (2023) elevated Pluteusseticepsvar.cystidiosus to *P.cystidiosus* based on specimens from the USA, Canada, Japan, and Russia. The present study reports *P.cystidiosus* as a new record in China. There was almost complete overlap in morphological variation between those reported in the present study and the holotype specimen. Both grow in temperate/cold-temperate forests. However, the basidiospores of the species in the present study were slightly larger, measuring (–5.0) 5.5–6.0 (–6.5) × (–4.5) 5.0–5.5 µm, while those of the holotype specimen were smaller, measuring 4.5–5.5 (–6.2) × 3.5–5.0 µm.

**Figure 4.**
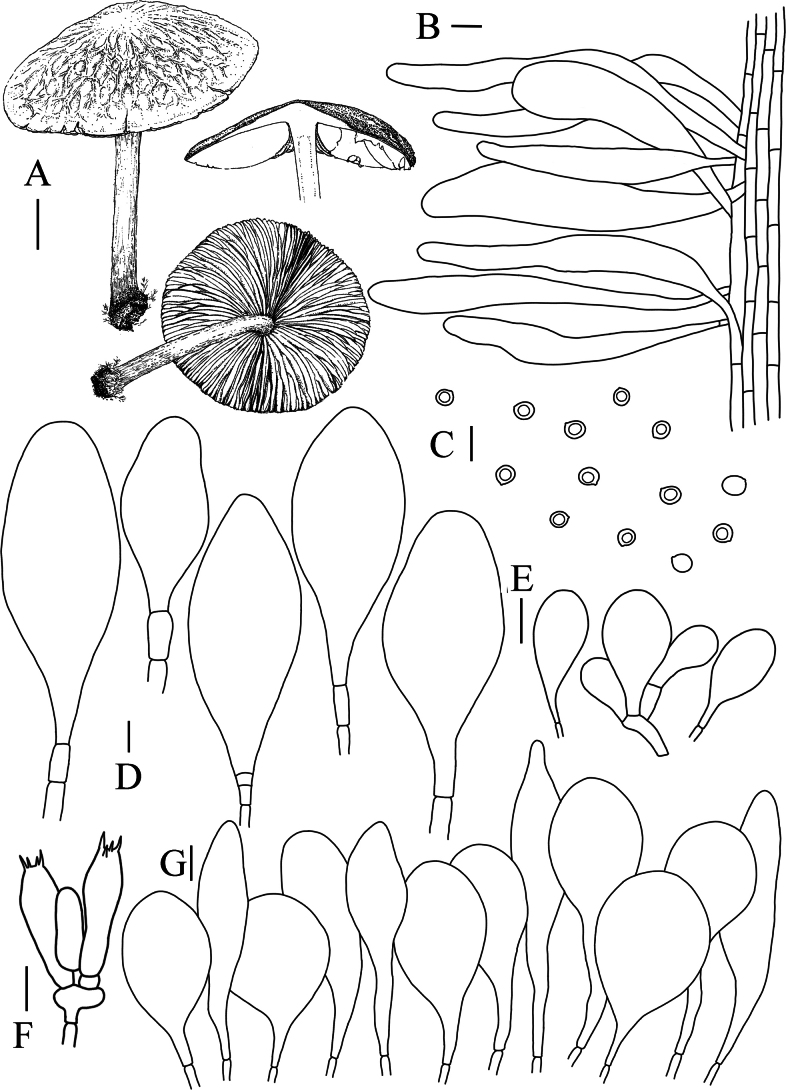
**A** Macroscopic characteristics of *Pluteuscystidiosus***B** caulocystidia **C** basidiospores **D** pleurocystidia **E** cheilocystidia **F** basidia **G** pileipellis. Scale bars: 1 cm (**A**); 10 µm (**B–G**).

The phylogenetic tree also supports the results of our morphological study, showing that our specimens are clustered in the same branch as those from the USA and Russia, with a support ratio of 1/100.

### ﻿ Pluteus chrysophlebius

Taxon classificationFungiAgaricalesPluteaceae

(Berk. & M.A. Curtis) Sacc., Syll. fung. (Abellini) 5: 678 (1887)

CA763193-D2E7-5972-B55A-0E3C5E0EAEAA

Figs 2E–F , 5

Agaricus chrysophlebius Berk. and M.A. Curtis 1859. Syn.

#### Description.

Basidiomata medium. Pileus 15–22 mm in diameter, surface not spreading, irregularly pitted, smooth, central part umbo, wrinkled or veined, yellow to bright yellow (5.0Y 9/12-5.0Y 9/20), with a hyaline stripe in the central part 3/4 of the way toward the margin, margin entire. Context yellowish (5.0Y 9/8), odor inconspicuous. Lamellae yellow to brownish yellow (5.0Y 9/6- 5.0Y 9/8), free, dense, thick, unequal, ventricose, 6–8 mm wide. Stipe 25–42 mm long, 4–6 mm wide, cylindrical, slightly thicker at the base, fibrous, bright yellow to yellow (5.0Y 9/10-5.0Y 9/18), smooth, with white tomentose dense cilia at the base. Odorless. Spore prints pink.

Basidiospores [90, 3, 1] 5.5–6.0 × (–4.5) 5.0–5.5 μm, avL × avW = 6.0 × 5.0 µm, Q = 1.09–1.20–1.33 μm, avQ = 1.20 μm, globose, subglobose, slightly pinkish, smooth, thinly walled, non-dextrinoid, partially containing one droplet or irregular inclusions. Basidia 23–34 × 7–11 μm, clavate, thin-walled, 4-sterigmate, and hyaline in KOH. Pleurocystidia scattered, 52–78 × 15–24 μm, broad and long-necked vase-like, partly with a long neck, neck with inclusions, thin-walled, smooth, and hyaline in KOH. Chilocystidia abundant, clustered, smaller, 45–66 × 14–21 μm, similar to pleurocystidia, long-necked vase-shaped to fusiform, thin-walled. Lamellar trama divergent. Pileipellis an euhymeniderm of spheropedunculate and subglobose elements 28–67 × 18–41 μm, with brown or light brown, at the center brown to dark brown. Stipitipellis a cutis, hyphae 5–9 μm wide, hyaline, non-gelatinous, thin-walled. Caulocystidia absent. Clamp connections absent in all tissues.

**Figure 5.**
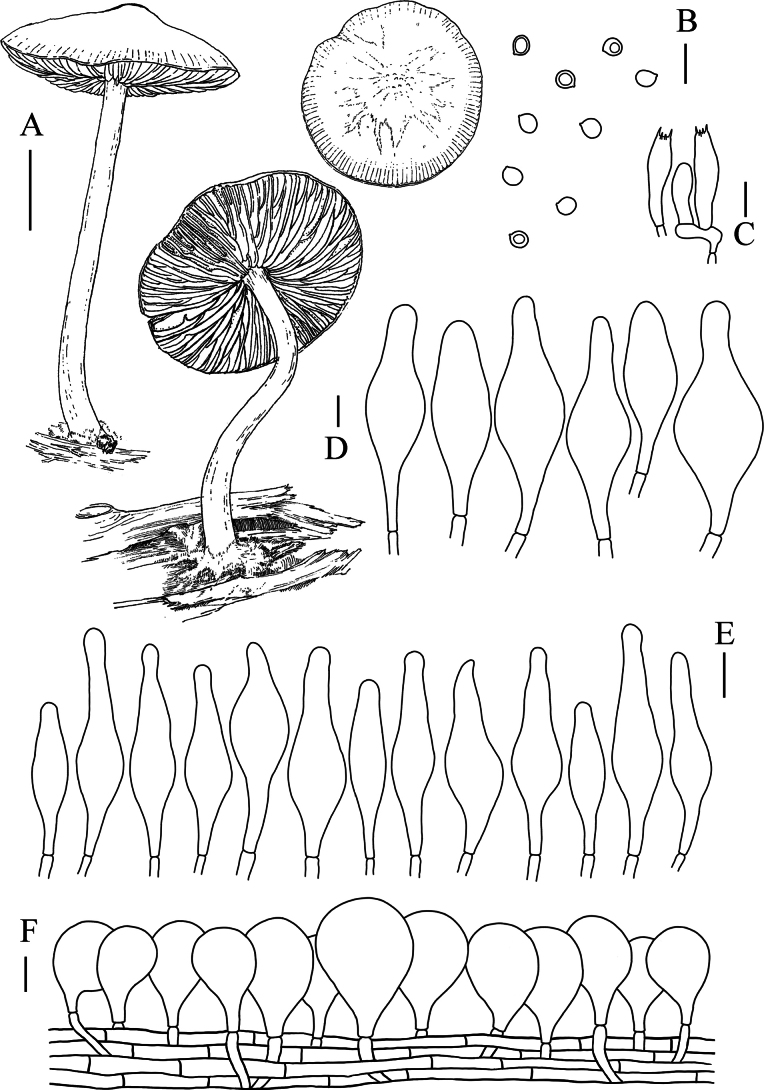
**A** macroscopic characteristics of *Pluteuschrysophlebius***B** basidiospores **C** basidia **D** pleurocystidia **E** cheilocystidia **F** pileipellis. Scale bars: 1 cm (**A**); 10 µm (**B–G**).

#### Ecology.

Solitary on decaying wood in mixed coniferous forests.

#### Distribution.

North America, South America.

#### Additional specimens examined.

China. Heilongjiang Province, Liangshui National Nature Reserve. 47°11'22.24"N, 128°47'89.11"E, 24 June 2019, D.Z. Guo, FJAU 66561 (ITS: OR994065, TEF1-α: PP062824).

#### Note.

*Pluteuschrysophlebius* was first reported in China. It can be distinguished from other yellow-pileus species such as *P.admirabilis* (Peck) Peck, *P.aurantiacus* Murrill, *P.melleus* Murrill, and *P.rugosidiscus* Murrill by its yellowish pileus and stipe, as well as its bald pileus texture (Minnis and Sundberg 2010; Malysheva et al. 2016). The phylogenetic analysis also supports the differentiation of species.

In the phylogenetic tree, *P.chrysophlebius* formed a cluster with TNSF12383 and TNSF12388 in Asia and was sister to SF10-SF12 in the United States, with strong support for both clades.

### ﻿ Pluteus romellii

Taxon classificationFungiAgaricalesPluteaceae

(Britzelm.) Lapl., Dict. iconogr. champ. sup. (Paris): 533 (1894)

8F85953E-D614-57E5-BC99-473A8CC34B56

Figs 2G–I , 6

Agaricus romellii Britzelm., Hymenomyceten aus Südbayern VIII: 5 (1891). Syn.

#### Description.

Basidiomata medium to large. Pileus 20–56 mm broad, compressed hemispherical to spreading, surface with vein-like projections extending to the pileus margin, often with striated dehiscence, with a greasy or almost waxy texture, brown to yellowish-brown (7.5YR 8/8-7.5YR 6/12), margins wavy dehiscence with translucent-striate. Context light yellow (7.5YR 8/12), odorless, 2–3 mm thick. Lamellae yellowish (10.0YR 8/10), free, medium dense, unequal, entire, ventricose, 5–7 mm wide. Stipe 26–41 mm long and 4–8 mm wide, cylindrical, slightly thicker at the base, fibrous, upper part of the stipe white to yellowish (10.0YR 9/8-10.0YR 7/12), smooth, lower part of the stipe with white tomentum, yellow to yellow-brown (10.0YR 8/8-10.0YR 8/12). Odorless. Spore print pale pink.

Basidiospores [120, 4, 2] 7.0–7.5 (–8.0) × 6.0–6.5 µm, avL × avW = 7.0 × 6.0 µm, Q = 1.07–1.25~1.33 µm, avQ = 1.16 µm, globose, subglobose to ellipsoid, transparent to slightly pinkish, smooth, and thin-walled, non-dextrinoid, partially containing one droplet or irregular inclusions. Basidia 27–32 × 8–10 μm, clavate, thin-walled, 4-sterigmate, and hyaline in KOH. Pleurocystidia abundant, scattered, 55–102 × 22–36 μm, rod-shaped or subcylindrical, fusiform, with neck and apical part broader and obtuse, thinly walled, smooth, and hyaline in KOH. Cheliocystidia abundant, clustered, 41–79 × 18–29 μm, pyriform or similarly pleurocystidia shape, thin-walled. Lamellar trama divergent. Pileipellis an euhymeniderm of spheropedunculate and subglobose elements 25–48 × 23–35 μm, with brown or light brown, at the center brown to dark brown. Stipitipellis a cutis, hyphae 6–10 μm wide, hyaline, non-gelatinous, thin-walled. Caulocystidia absent. Clamp connections absent in all tissues.

**Figure 6.**
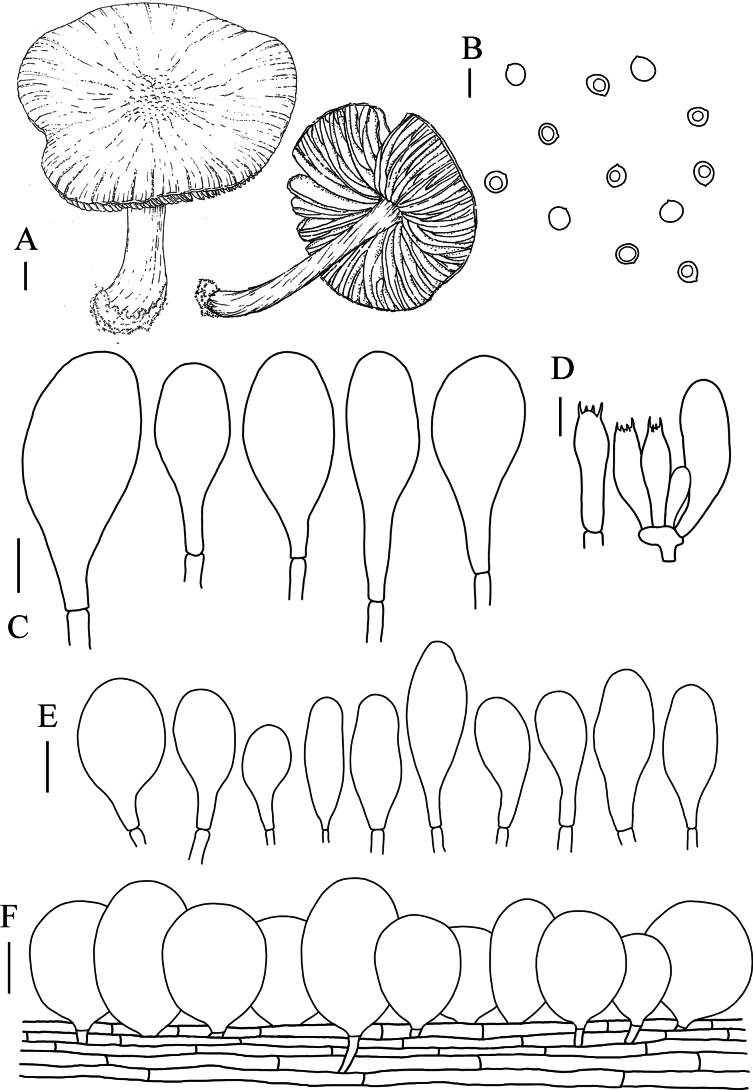
**A** macroscopic characteristics of *Pluteusromellii***B** basidiospores **C** pleurocystidia **D** basidia **E** cheilocystidia **F** pileipellis. Scale bars: 1 cm (**A**); 10 µm (**B–E**); 20 µm (**F**).

#### Ecology.

Solitary to scattered on decaying wood in coniferous forests (*Piceaschrenkiana* Fisch.).

#### Distribution.

Europe, Americas, East Asia, Africa.

#### Additional specimens examined.

China. Xinjiang Uygur Autonomous Region, Ili Kazakh Autonomous Prefecture, Tekes County, Jongkushtai Village, 43°12'26.61"N, 81°91'97.21"E, alt. 2139 m, 10 July 2022, Z.X. Qi, J.J. Hu, and B. Zhang, FJAU 66558 (ITS: OR994057, TEF1-α: PP062827). China. Xinjiang Uygur Autonomous Region, Ili Kazakh Autonomous Prefecture, Tekes County, Jongkushtai Village, 43°15'22.61"N, 81°75'90.21"E, alt. 2147 m, 11 July 2022, Z.X. Qi, J.J. Hu, and B. Zhang, FJAU 66559 (ITS: OR994061, TEF1-α: PP062828).

#### Note.

Initially, the description of *Pluteusromellii* was rather vague (Britzelmayr 1891), stating that *P.romellii* was similar to *P.nanus* (Pers.) P. Kumm, with spores measuring 6–7 μm, and found growing in the soil of Bavaria. It is now widely acknowledged that *P.romellii* is characterized by a brown pileus, yellow stipe, and the absence of elongated elements in the pileipellis. This species is placed on the phylogenetic tree in subsect. Eucellulodermini under sect. Celluloderma (Orton 1986; Vellinga 1990; Ševcíková et al. 2023). Here, our description of the *P.romellii* is consistent with the commonly accepted characterization. Phylogenetic analysis shows that it clustered with the epitype (BRNM 761731) with strongly supported (99/0.98).

### ﻿Key to the reported species of Pluteussect.Celluloderma in China

**Table:** 

1	Pileipellis consists of spheropedunculate cells and elongated cystidioid elements	**2**
–	Pileipellis consists of spheropedunculate cells without elongated cystidioid elements	**7**
2	With caulocystidia	**3**
–	Without caulocystidia	**6**
3	With pleurocystidia	**4**
–	Without pleurocystidia	***Pluteuscinnabarinus***
4	Cheilocystidia with short to long mucronate at the apex	***Pluteusaurantioruber***
–	Cheilocystidia without short to long mucronate at the apex	**5**
5	Pleurocystidia larger, measuring 35–73 (–82) × 11–31 µm	***Pluteuscystidiosus***
–	Pleurocystidia smaller, measuring 36–51 × 13.4–24 µm	***Pluteuspodospileus***
6	Pileus middle reticulate elevated, radially rugose	***Pluteusthomsonii***
–	Pileus brown with stripes extending to the margins	***Pluteusstriatus***
7	Pileipellis consists of globular, obpyriform, or spheropedunculate cells	**8**
–	Pileipellis consists of without globular, obpyriform, or spheropedunculate cells	**16**
8	Grows on rotting wood	**9**
–	Grows on non-rotting wood	***Pluteusaletaiensis***
9	Pileus, stipe bright-colored	**10**
–	Pileus, stipe not bright-colored	**13**
10	Pileus middle folded, groove-like striate	***Pluteuschrysophaeus***
–	Pileus middle non-folded, groove-like striate	**11**
11	Pileus bright red or orange-red	***Pluteusaurantiorugosus***
–	Pileus non-bright red to orange-red	**12**
12	Pileus smooth, widely distributed in North America	***Pluteuschrysophlebius***
–	Pileus goose-yellow, margin striate	***Pluteusadmirabilis***
13	Basidiomata small	***Pluteusnanus***
–	Basidiomata non-small	**14**
14	Lamellae edged with a powdery creamy material	***Pluteuspulverulentus***
–	Lamellae edged without a powdery creamy material	**15**
15	Pleurocystidia with neck and broad, blunt apex	***Pluteusromellii***
–	Pileus teal brown, dark cinnamon-colored, with black ribbed veins or wrinkles	***Pluteusphlebophorus***
16	Grows on rotting wood	**17**
–	Grows on non-rotting wood	***Pluteusbrunneodiscus***
17	Pileus margin with hyaline stripes	**18**
–	Pileus margin without hyaline stripes	**19**
18	Cheilocystidia with mucronate at the apex	***Pluteuspallidus***
–	Cheilocystidia without mucronate at the apex	***Pluteusbrunneoalbus***
19	Pileus with dark brown frosting powder, radially dehiscent to margins	***Pluteusdiettrichii***
–	Pileus surface squarrose, stipe with surface covered by caulocystidia elements	***Pluteussquarrosus***

## ﻿Discussion

Singer (1986) and Vellinga and Schreurs (1985) classified sect. Celluloderma into two subsections: subsect. Celluloderma and subsect. Mixtini. However, subsequent systematic analyses of sect. Celluloderma did not have a high level of support from internal topology analysis, leading to the conclusion subsect. Eucellulodermini and subsect. Mixtini should not conform to natural taxonomy. Singer (1986) proposed that species with non-metuloid cystidia, a cutis, and trichodermal pileipellis should be classified in the sect. Hispidoderma. Vellinga and Schreurs (1985) proposed sect. Villosi on the basis of a cutis-like pileipellis and non-metuloid cystidia. However, in the ephebeus clade, there are *P.ephebeus* from Europe and P.riberaltensisvar.conquistensis from the USA. These species should be placed in sect. Hispidoderma and classified based on the pileipellis, but molecular results indicate that it belongs to sect. Celluloderma. In the phylogenetic tree, it is the sister group to *P.fenzlii*, *P.mammillatus*, and some species have a partial veil. *P.brunneodiscus* in the ephebeus clade in the present study, which has non-metuloid cystidia and pileipellis as a cutis, shares their views with Vellinga and Schreurs (1985). The phylogenetic tree also exhibits a high level of support. Further research is needed to restore these species to sect. Villosi.

The presence of a partial veil in *P.aurantiorugosus*, P.aurantiorugosusvar.aurantiovelatus, *P.fenzlii*, and *P.mammillatus* suggests that the occurrence or nonoccurrence/ lack of the partial veil in the evolutionary history of *Pluteus* occurred independently. As stated by Singer states (Singer 1958; Minnis and Sundberg 2010; Justo et al. 2011a, 2011b; Vizzini and Ercole 2011), this characteristic is homoplasic and unsuitable for the natural classification of these fungi at the supraspecific rank.

## Supplementary Material

XML Treatment for Pluteus brunneodiscus

XML Treatment for Pluteus cystidiosus

XML Treatment for Pluteus chrysophlebius

XML Treatment for Pluteus romellii

## ﻿Additional information

### Author ORCIDs

Zheng-Xiang Qi https://orcid.org/0000-0002-0037-9407

Ke-Qing Qian https://orcid.org/0000-0002-4627-5240

Bo Zhang https://orcid.org/0000-0001-9508-8188

### Data availability

All of the data that support the findings of this study are available in the main text.
